# Inverse association between serum iron levels and Hashimoto’s thyroiditis in United States females of reproductive age: analysis of the NHANES 2007–2012

**DOI:** 10.3389/fnut.2024.1410538

**Published:** 2024-10-01

**Authors:** Liang Zhang, Yibing Li, Liu Yang, Zhixiong Luo, Zhaoyu Wu, Jingbo Wang, Siyuan Qin, Fei Ren, Tianyuan Hu

**Affiliations:** Department of Nuclear Medicine, Honghui Hospital, Xi'an Jiaotong University, Xi’an, China

**Keywords:** serum iron, Hashimoto’s thyroiditis, female, cross-sectional study, NHANES

## Abstract

**Purpose:**

Hashimoto’s thyroiditis (HT) is a significant public health concern, particularly among females. While existing studies have explored the correlation between serum iron levels and HT, limited research has specifically focused on this association in reproductive-age females. Our study aims to investigate the relationship between serum iron and HT.

**Methods:**

Using data from the National Health and Nutrition Examination Survey (NHANES) database (2007–2012), we employed weighted multivariate logistic regression models, an XGBoost model, and smooth curve fitting. We assessed the correlation between serum iron and HT and examined linear and non-linear relationships with thyroid peroxidase antibodies (TPOAb) and thyroglobulin antibodies (TgAb).

**Results:**

Among 2,356 participants, each unit increase in serum iron was associated with a 43% reduced risk of HT (Odds Ratios (OR) 0.574; 95% Confidence Interval (CI) 0.572, 0.576). Quartile analysis confirmed these effects. The XGBoost model identified serum iron as the most significant variable correlated with HT. Smooth curves revealed a linear association between log2-transformed serum iron and HT. Additionally, log2-transformed serum iron inversely correlated with TPOAb levels (*β* −15.47; 95% CI -25.01, −5.92), while a non-linear relationship was observed with TgAb.

**Conclusion:**

Our study reveals that in reproductive-age women, every unit increase in serum iron is associated with a 43% lower risk of HT, demonstrating an inverse relationship. Additionally, serum iron exhibits a negative correlation with TPOAb and a non-linear association with TgAb.

## 1 Introduction

Hashimoto’s Thyroiditis (HT), also known as chronic lymphocytic thyroiditis or autoimmune thyroiditis, is a widespread organ-specific autoimmune disease first documented by Haraku Hashimoto in 1912 and is one of the most common human ailments (1, 2). It is characterized by an enlarged thyroid, lymphocytic infiltration, and the production of antibodies against thyroid antigens. These characteristics trigger an immune reaction, which increases the risk of thyroid malignancies (3, 4). Notably, female HT patients have a 30% higher likelihood of developing papillary thyroid carcinoma (PTC) compared to their male counterparts (5). The incidence of HT ranges from 27 to 448 cases per 100,000 people per year, varying by study design and geographic area (6). Over recent decades, the incidence of HT has seen a rapid upsurge (7). The incidence of HT is higher in women, with rates varying between 4.8 to 25.8%, compared to men who have a prevalence of 0.9 to 7.9%. The likelihood of being diagnosed with HT escalates with age, often surfacing during the prime reproductive years (8–10). Consequently, HT has become a significant public health concern, particularly for the female population.

Despite its high prevalence, the pathogenesis of HT is still not fully understood. The disease arises from a complex interplay of genetic susceptibility, environmental triggers, and immune dysregulation (11–13). Extensive research has delved into the influence of trace elements and environmental contributors. Trace elements, including iodine, iron, selenium, zinc, copper, calcium and cadmium, are crucial for thyroid metabolism, function, and hormone synthesis. Abnormalities in these elements can affect thyroid autoimmunity, tumorigenesis, and iodine uptake (14–16).

Iron is essential for thyroid metabolism, particularly in the context of HT. It is a key component in the production of thyroid hormones T3 and T4, as it is integral to the functioning of Thyroid Peroxidase (TPO), an iron-dependent enzyme (15, 17–19). In addition to its role in thyroid metabolism, iron levels in the body can be influenced by other conditions often seen in HT patients. HT patients often suffer from co-morbid conditions like celiac disease and autoimmune gastritis, which are major causes of iron deficiency (20–25). Celiac disease impairs iron absorption, while autoimmune gastritis, characterized by antibodies against parietal cells and intrinsic factor, can cause severe atrophic gastritis, low stomach acid, and chronic iron deficiency, affecting the absorption of dietary non-heme iron (26, 27). While it has been reported that patients with subclinical hypothyroidism due to HT often exhibit lower iron levels and higher rates of iron deficiency compared to healthy individuals (28), the association between serum iron levels and HT in females, particularly females of reproductive age, is under-researched in cross-sectional studies.

Therefore, the aim of this research is to further investigate the correlation between serum iron levels and HT in females of reproductive age, we have selected the age range of 15 to 44 years as a representative sample. Understanding this correlation could potentially lead to new strategies for managing HT in this demographic. Our objective is to gain a more comprehensive understanding of this intricate interaction. Through the execution of a comprehensive cross-sectional study utilizing the National Health and Nutrition Examination Survey (NHANES) database (2007–2012), our aim is to provide further insights into this intricate relationship.

## 2 Materials and methods

### 2.1 Data sources and study population

NHANES, conducted by the United States National Center for Health Statistics, is a crucial tool for health and nutritional research. It employs a comprehensive methodology to assess the health and nutritional status of non-institutionalized United States residents aged over two, with oversampling of certain subgroups to ensure data precision. The survey collects demographic, socioeconomic, health, and dietary data through questionnaires, interviews, and physical examinations, conducted biennially since 1999. This allows for longitudinal tracking of health changes. The data, collected through laboratory tests, physical examinations, and interviews, is vital for epidemiological studies, disease prevalence assessment, risk factor identification, and evaluation of the association between nutrition and health. The rigorous approach of NHANES ensures the reliability and validity of the research conducted using its data. The findings significantly contribute to public health policy development and advance national health knowledge (29). The protocol for the survey has received approval from the Ethics Review Committee of the National Center for Health Statistics, and informed consent is provided by all the participants (30).

**Figure 1:**
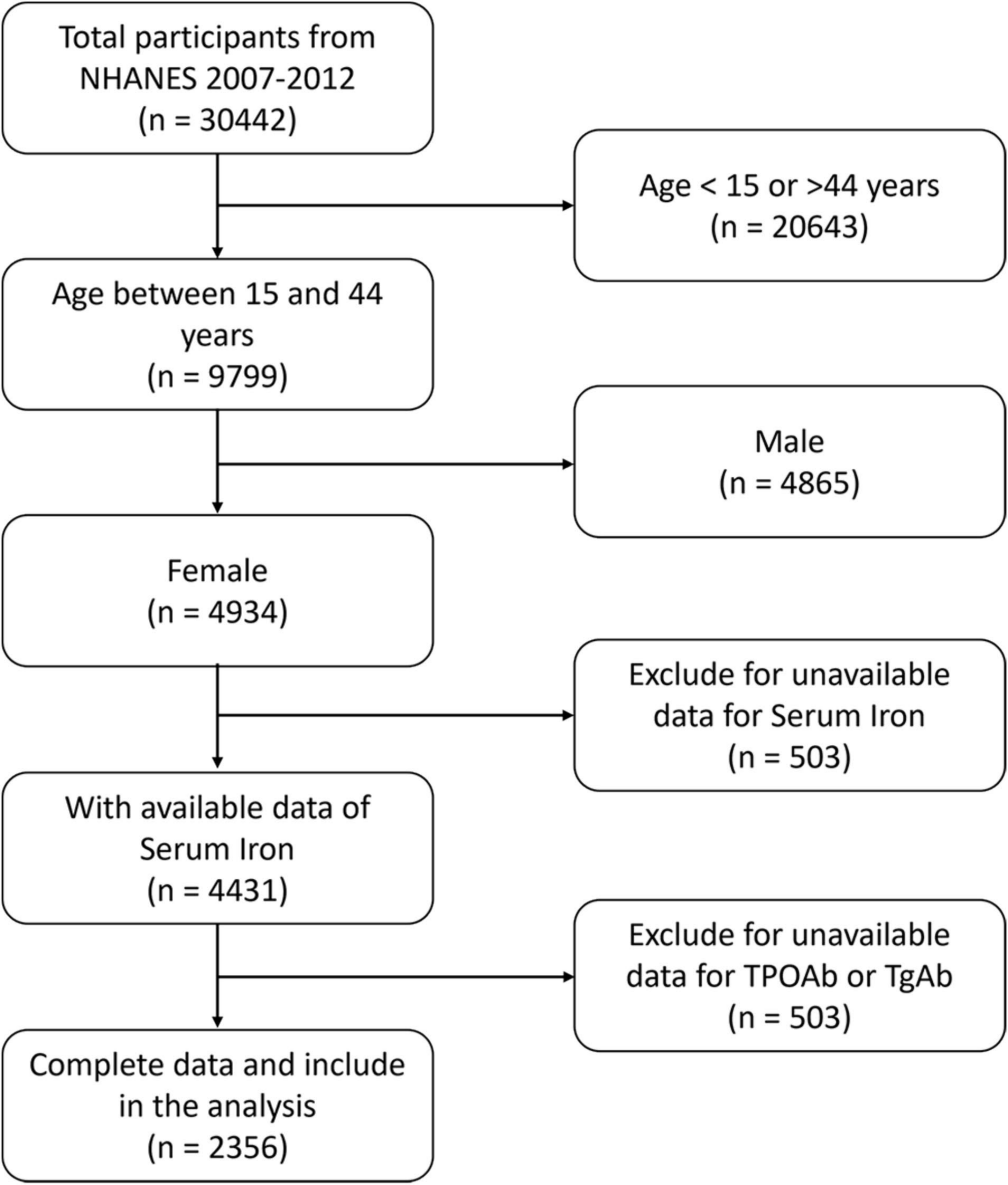
Flow chart of the studying participants’ selection from the NHANES 2007–2012.

While all NHANES datasets provide data on serum iron, it is only the 2007–2012 dataset (which comprises three research cycles, each spanning 2 years) that includes comprehensive thyroid function data. Consequently, this analysis employed data from the 2007–2012 dataset, with a specific emphasis on participants who had exhaustive data for both the predictors, namely serum iron, and the outcomes, specifically HT. The study excluded individuals who were under 15 or over 44 years of age (*n* = 20,643), males (*n* = 4,865), and participants who had missing data on serum iron (*n* = 503), or thyroid peroxidase antibodies (TPOAb) or thyroglobulin antibodies (TgAb) (*n* = 2075). Consequently, the study incorporated a total of 2,356 participants out of 30,442, who adhered to the inclusion criteria (Figure 1). Furthermore, the study was conducted in compliance with the revised 2013 Declaration of Helsinki.

### 2.2 Measurement of serum iron

Serum samples in the NHANES studies underwent processing and storage, before being sent to the Collaborative Laboratory Services for examination, in accordance with the guidelines in the NHANES Laboratory/Medical Technologists Procedures Manual (LPM). The vials were kept in suitable frozen conditions (−30°C) until they were dispatched to the National Center for Environmental Health for testing. During 2007 to 2008, both the LX20 and DcX800 timed-endpoint methods were employed for the measurement. However, from 2009 to 2012, only the DcX800 method was put into use. In these methods, iron is detached from transferrin, reduced to the ferrous state, and complexed with the FerroZine Iron Reagent. The alteration in absorbance at 560 nm, which is in direct proportion to the iron concentration, is then observed.

### 2.3 Examination of HT

HT, identified as the outcome variable, is diagnosed by the presence of thyroid autoantibodies. The titers of TgAb and TPOAb are determined using a sequential two-step immunoenzymatic ‘sandwich’ assay. Individuals with a TgAb titer of 115.0 IU/mL or higher and/or a TPOAb titer of 9.0 IU/mL or higher are considered positive (31).

### 2.4 Covariates

Based on previous similar studies and clinical experience, we identified the following variables as potential confounders in this study because they may affect our statistical results: age, race/ethnicity, education level, marital status, ratio of household income to poverty, and body mass index (BMI, kg/m2). Laboratory data included thyroid-stimulating hormone (TSH, Miu/L), free triiodothyronine (FT3, pg./Ml), free thyroxine (FT4, pmol/L), thyroglobulin (TG, ng/ml), TPOAb (IU/ml), TgAb (IU/ml), glycosylated hemoglobin A1c (HbA1c, %), hemoglobin (g/Dl), hematocrit (%), mean cell volume (MCV, Fl), triglycerides (mg/Dl), cholesterol (mg/Dl), high-density lipoprotein cholesterol (HDL-C, mg/Dl), low-density lipoprotein cholesterol (LDL-C, mg/Dl), urinary iodine (ug/L), albumin (g/Dl), serum uric acid (mg/Dl), creatinine (mg/Dl), phosphorus (mg/Dl), total calcium (mg/Dl), and blood urea nitrogen (mg/Dl). The extracted questionnaire data encompassed aspects such as alcohol intake, smoking, hypertension, diabetes, and physical activity.

BMI is calculated by dividing the weight in kilograms by the square of the height in meters. Race/ethnicity was categorized as Mexican American, Other Hispanic, Non-Hispanic White, Non-Hispanic Black, and other races. Marital status was categorized as married/living with partner, never married, and other. Education level was classified as high school or below and above high school. Smoking was characterized as having consumed more than 100 cigarettes in one’s lifetime (32). Alcohol intake was characterized as having a minimum of 12 alcoholic beverages per year. Hypertension was characterized as having a systolic blood pressure (SBP) of ≥140 or/and diastolic blood pressure (DBP) of ≥90 mmHg or a history of hypertension (33). Diabetes was characterized as a self-reported medical diagnosis of diabetes, the usage of antidiabetic medications or insulin, a HbA1c level of ≥6.5%, or a fasting glucose level of ≥126 mg/Dl (34, 35). Physical activity was classified as ‘Yes’ for those who participate in vigorous or moderate recreational activities, and ‘No’ for those who do not participate in moderate recreational activities (36).

### 2.5 Statistical analysis

Statistical analysis was performed using the Statistical Package for the Social Sciences (SPSS, version 26.0) and R software (version 4.4.2). The analysis was performed in line with the survey methods and the guidelines stipulated by NHANES. Continuous variables with normal distribution are expressed as weighted mean ± standard deviation (Mean ± SD), with differences analyzed using the independent t-test. For skewed distributions, continuous variables are presented as weighted median with interquartile range (IQR), and differences between groups are analyzed using the Kruskal-Wallis rank sum test. Categorical variables are represented as percentages, with differences analyzed using the chi-squared test. Serum iron concentrations were transformed using a logarithm base 2 (Log2) for subsequent analyses due to their skewed distribution. Serum iron concentrations were also divided into four quartiles. The objective of the study was to delve into the relationship between serum iron and the onset of HT in a specific population. A multivariate logistic regression model was built to evaluate the odds ratios (OR) and 95% Confidence Interval (CI) between serum iron and HT. This model was split into three parts: Unadjusted Model (Model 1): Adjusted for none. Minimally Adjusted Model (Model 2): Adjusted for age and race/ethnicity. Fully Adjusted Model (Model 3): Included the variables from Model 2 and was further adjusted for education level, marital status, poverty income ratio, BMI, alcohol intake, smoking, FT3, FT4, TSH, physical activity, hypertension, diabetes, Iodine, Calcium. Subgroup analyses were carried out on variables including age, race/ethnicity, education level, marital status, alcohol intake, smoking, physical activity, poverty income ratio, hypertension, and diabetes to assess potential effect modification. Generalized Additive Models (GAMs) were utilized to test potential non-linear relationships of serum iron with HT, TPOAb and TgAb, adjusting the GAMs for the same covariates as linear regression models. Statistical significance was set at *p* < 0.05.

## 3 Results

### 3.1 Characteristics of participants

Table 1 showcases the characteristics of the NHANES participants included in our analysis. The study cohort is composed of females of reproductive age with an average age of 29.65 years. Table 1 provides a comprehensive overview of sociodemographic data, laboratory test outcomes, dietary details, physical activity data derived from questionnaires, and comorbidity statistics for the participants. Of the total cohort, 247 individuals were diagnosed with HT, representing 10.48% of the total cohort. Compared to individuals without HT, those diagnosed with HT are typically older and have a higher poverty-income ratio (*p* < 0.05). Moreover, HT patients exhibited elevated levels of TSH, TPOAb, and TgAb, alongside reduced serum iron and urinary iodine levels when compared to their non-HT counterparts (*p* < 0.05). A greater prevalence of diabetes mellitus was noted among HT patients (*p* < 0.05). While race/ethnicity and marital status initially indicated significant differences, further analysis between the two groups revealed no significant disparities.

**Table 1:** Baseline characteristics of selected participants from the NHANES 2007–2012.

Characteristic	Total (*n* = 2,356)	Non-Hashimoto’s thyroiditis (*n* = 2,109)	Hashimoto’s thyroiditis (*n* = 247)	*p* value
Age, mean ± SD (years)	29.65 ± 8.90	29.41 ± 8.88	31.49 ± 8.86	0.003
Race/ethnicity (%)				<0.001
Mexican American	10.2	10.3	9.2	
Other Hisapanic	6.8	6.7	7.0	
Non-Hispanic White	63.3	62.1	72.6	
Non-Hispanic Black	12.1	13.3	3.4	
Other Race	7.7	7.6	7.8	
Education level (%)				0.233
High school or below	45.4	46.0	41.3	
Above high school	54.6	54.0	58.7	
Marital status (%)				0.032
Married or living with a partner	62.3	61.0	71.4	
Never married	27.1	28.1	20.0	
Other	10.6	10.9	8.6	
Poverty income ratio, median (IQR)	2.42 (3.17)	2.33 (3.17)	2.96 (2.86)	0.007
BMI, median (IQR) (kg/m2)	25.71 (9.23)	25.72 (9.26)	25.60 (9.14)	0.677
Alcohol intake (%)				0.944
Yes	74.1	74.1	73.8	
No	25.9	25.9	26.2	
Smoking (%)				0.419
Yes	35.9	36.3	32.9	
No	64.1	63.7	67.1	
Physical activity (%)				0.098
Yes	59.4	58.7	65.0	
No	40.6	41.3	35.0	
Serum iron, median (IQR) (mg/dL)	73.00 (48.00)	74.00 (48.00)	68.00 (22.34)	0.045
FT3, median (IQR) (pg/mL)	3.20 (0.42)	3.20 (0.41)	3.10 (0.40)	0.007
FT4, median (IQR) (pmol/L)	10.30 (2.00)	10.30 (2.00)	10.30 (1.77)	0.150
TSH, median (IQR) (mIU/L)	1.46 (1.17)	1.39 (1.06)	2.13 (2.17)	<0.001
TG, median (IQR) (ng/mL)	10.30 (11.70)	10.43 (11.02)	8.79 (17.61)	0.038
TPOAb, median (IQR) (IU/mL)	0.60 (1.20)	0.60 (0.80)	98.39 (247.66)	<0.001
TgAb, median (IQR) (IU/mL)	0.60 (0.00)	0.60 (0.00)	1.31 (6.16)	<0.001
Albumin, mean ± SD (g/dL)	2.37 ± 0.10	4.19 ± 0.35	4.20 ± 0.34	0.767
Hemoglobin, mean ± SD (g/dL)	13.27 ± 1.23	13.30 ± 1.18	13.09 ± 1.53	0.084
Hematocrit, mean ± SD (%)	38.60 ± 3.42	38.68 ± 3.28	38.05 ± 4.32	0.058
MCV, mean ± SD (fL)	87.54 ± 6.29	87.63 ± 5.86	86.84 ± 8.84	0.247
Blood urea nitrogen, mean ± SD (mg/dL)	10.12 ± 3.48	10.07 ± 3.51	10.45 ± 3.24	0.117
Creatinine, mean ± SD (mg/dL)	0.72 ± 0.22	0.72 ± 0.22	0.70 ± 0.15	0.104
Phosphorus, mg/dL [mean (SD)]	3.84 ± 0.55	3.84 ± 0.55	3.82 ± 0.55	0.596
Triglycerides, median (IQR) (mg/dL)	91.00 (76.00)	91.00 (76.00)	89.93 (84.38)	0.921
HbA1c, mean ± SD (%)	5.27 ± 0.58	5.27 ± 0.57	5.31 ± 0.64	0.360
HDL-C, median (IQR) (mg/dL)	55.40 (20.52)	55.40 (20.52)	55.13 (21.65)	0.774
LDL-C, mean ± SD (mg/dL)	107.86 ± 32.79	107.63 ± 33.17	109.46 ± 30.09	0.602
Iodine, urine, median (IQR) (ug/L)	131.40 (161.42)	131.84 (162.05)	124.61 (150.35)	0.567
Total calcium, mg/dL [mean (SD)]	9.35 ± 0.34	9.35 ± 0.34	9.33 ± 0.32	0.332
Serum uric acid, mean ± SD (mg/dL)	4.50 ± 1.04	4.50 ± 1.03	4.54 ± 1.08	0.655
Total cholesterol, mean ± SD (mg/dL)	184.17 ± 37.78	183.78 ± 37.82	187.08 ± 37.45	0.276
Hypertension (%)				0.207
Yes	12.8	13.2	9.6	
No	87.2	86.8	90.4	
Diabetes (%)				0.014
Yes	2.9	2.6	5.8	
No	97.1	97.4	94.2	

### 3.2 The relationship between serum iron and HT

We constructed a multivariate logistic regression model to delve into the relationship between log2-transformed serum iron levels and HT, as depicted in Table 2. In the initial model (Model 1), which incorporated unadjusted variables, it was observed that each unit increment in serum iron was associated with a 35% decrease in the risk of HT (OR 0.652; 95% CI 0.650, 0.653), with a *p*-value for trend less than 0.05. When adjusted for age and race/ethnicity (Model 2), each unit increment in serum iron corresponded to a 39% reduction in HT risk (OR 0.606; 95% CI 0.605, 0.607), with the *p*-value for trend remaining below 0.05. In the fully adjusted model (Model 3), which accounted for all covariates, each unit increment in serum iron was linked to a 43% lower risk of HT (OR 0.574; 95% CI 0.572, 0.576), with the *p*-value for trend still less than 0.05. These findings suggest a robust inverse association between serum iron levels and the risk of HT, consistent across various levels of adjustment.

**Table 2:** The association between serum iron and Hashimoto’s thyroiditis.

Exposure	Model 1 OR (95% Cl)	*p* value	Model 2 OR (95% Cl)	*p* value	Model 3 OR (95% Cl)	*p* value
Serum Iron (log2 transformation)	0.652 (0.650,0.653)	<0.001	0.606 (0.605,0.607)	<0.001	0.574 (0.572,0.576)	<0.001
Q1(1.61,3.91)	Reference		Reference		Reference	
Q2(3.93,4.26)	0.901 (0.898,0.904)	<0.001	0.874 (0.872,0.877)	<0.001	0.977 (0.973,0.981)	<0.001
Q3(4.28,4.56)	0.915 (0.912,0.918)	<0.001	0.863 (0.860,0.866)	<0.001	0.951 (0.948,0.955)	<0.001
Q4(4.57,6.06)	0.491 (0.489,0.492)	<0.001	0.454 (0.453,0.456)	<0.001	0.354 (0.352,0.355)	<0.001
*p* trend	<0.001		<0.001		<0.001	

Model 1: adjusted for none. Model 2: age and race/ethnicity were adjusted. Model 3: age, race/ethnicity, education level, marital status, poverty income ratio, BMI, alcohol intake, smoking, FT3, FT4, TSH, physical activity, hypertension, diabetes, iodine, calcium were adjusted.

### 3.3 Stratified associations between serum iron and HT

**Figure 2:**
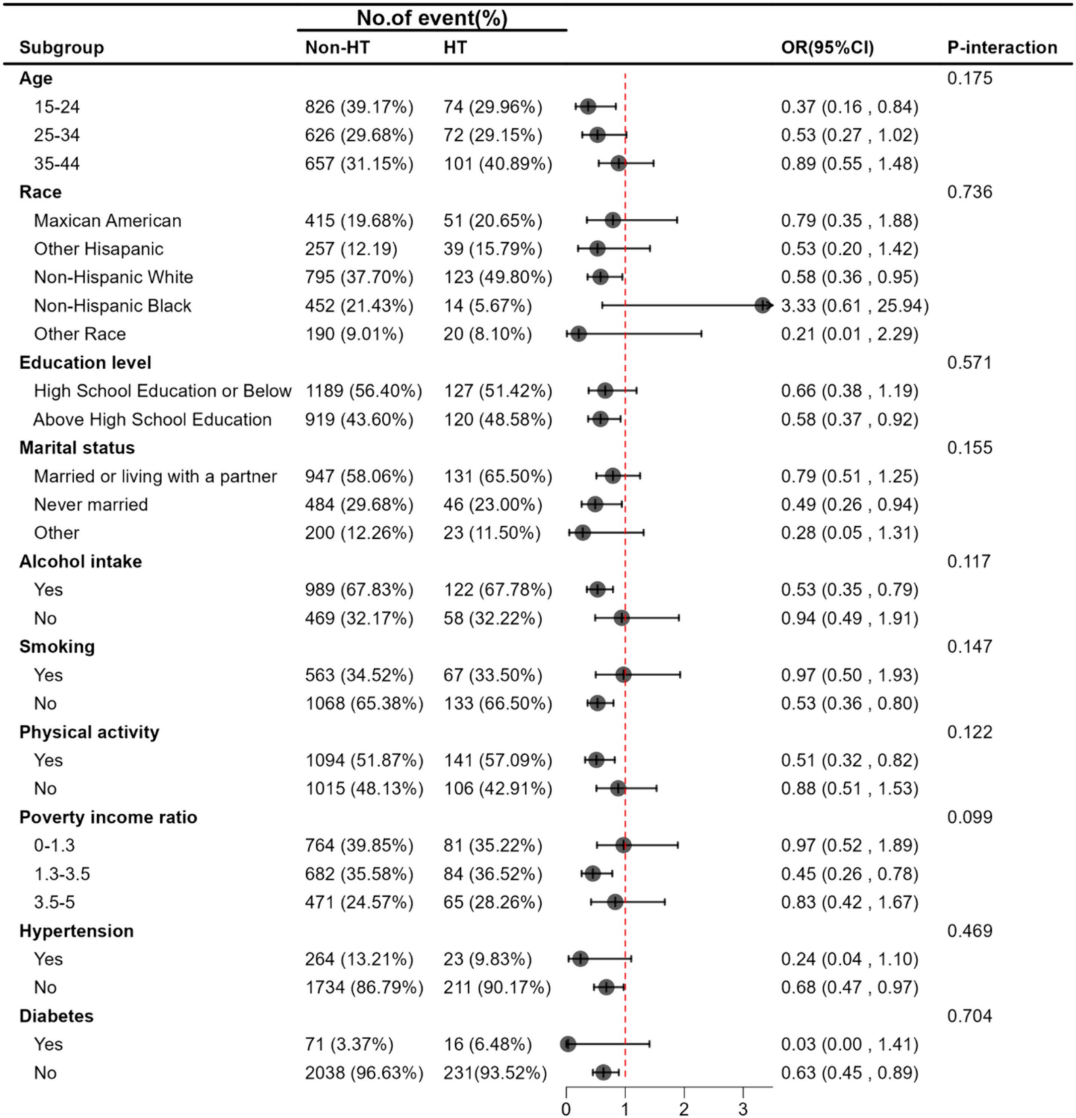
Stratified associations between Serum Iron and HT. Adjusted for age, race/ethnicity, education level, marital status, poverty income ratio, BMI, alcohol intake, smoking, FT3, FT4, TSH, physical activity, hypertension, diabetes, Iodine, Calcium.

Stratified analyses were performed using chosen variables (age, race/ethnicity, education level, marital status, alcohol intake, smoking, physical activity, poverty income ratio, hypertension, and diabetes) to further scrutinize the relationship between serum iron levels and HT. None of these variables significantly altered the association between log2-transformed serum iron and HT. This includes age (15–24, 25–34, 35–44, *p*-interaction = 0.175), race/ethnicity (Mexican American, other Hispanic, Non-Hispanic White, Non-Hispanic Black, other races; *p*-interaction = 0.736), education level (high school or below, above high school; *p*-interaction = 0.571), marital status (married or living with partner, never married, other; *p*-interaction = 0.155), alcohol intake (yes, no; *p*-interaction = 0.117), smoking status (yes, no; *p*-interaction = 0.147), physical activity (yes, no; *p*-interaction = 0.122), poverty income ratio (0–1.3, 1.3–3.5, 3.5–5, *p*-interaction = 0.099), hypertension (yes, no; *p*-interaction = 0.469), and diabetes (yes, no; *p*-interaction = 0.704), as shown in Figure 2.

### 3.4 Determination of importance of variables

**Figure 3:**
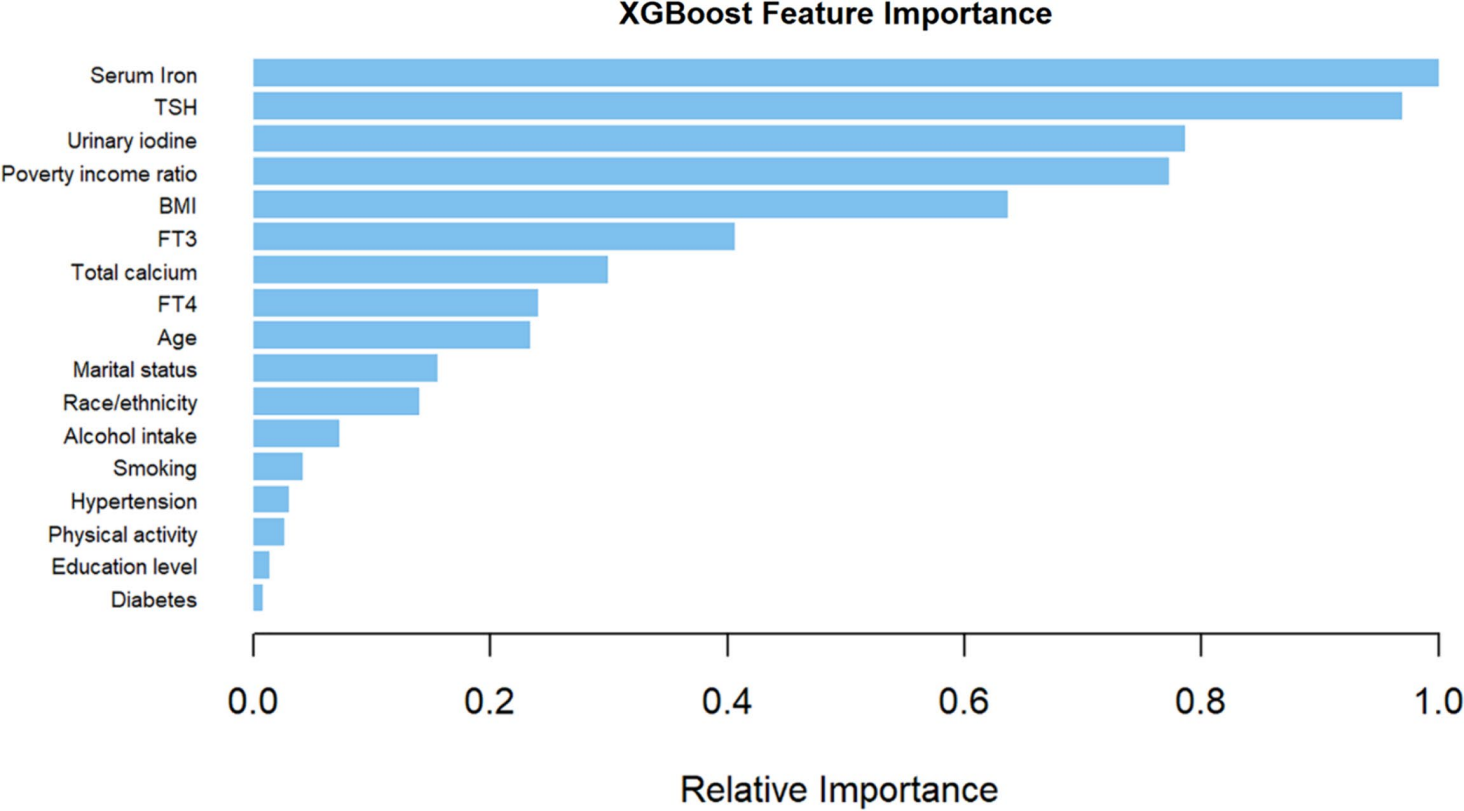
The relative importance was calculated for each variables using the XGBoost model.

The XGBoost model was employed to ascertain the relative importance of chosen variables, which include age, race/ethnicity, education level, marital status, poverty income ratio, BMI, alcohol intake, smoking habits, serum iron, FT3, FT4, TSH levels, physical activity, hypertension, diabetes, iodine intake, and calcium for HT. Based on their contributions in the XGBoost model, serum iron levels, TSH, and urinary iodine surfaced as the most crucial variables in the dataset (Figure 3). As a result, serum iron levels were used as the most relevant variable for constructing a smoothing curve model.

### 3.5 Sensitivity analysis

**Figure 4:**
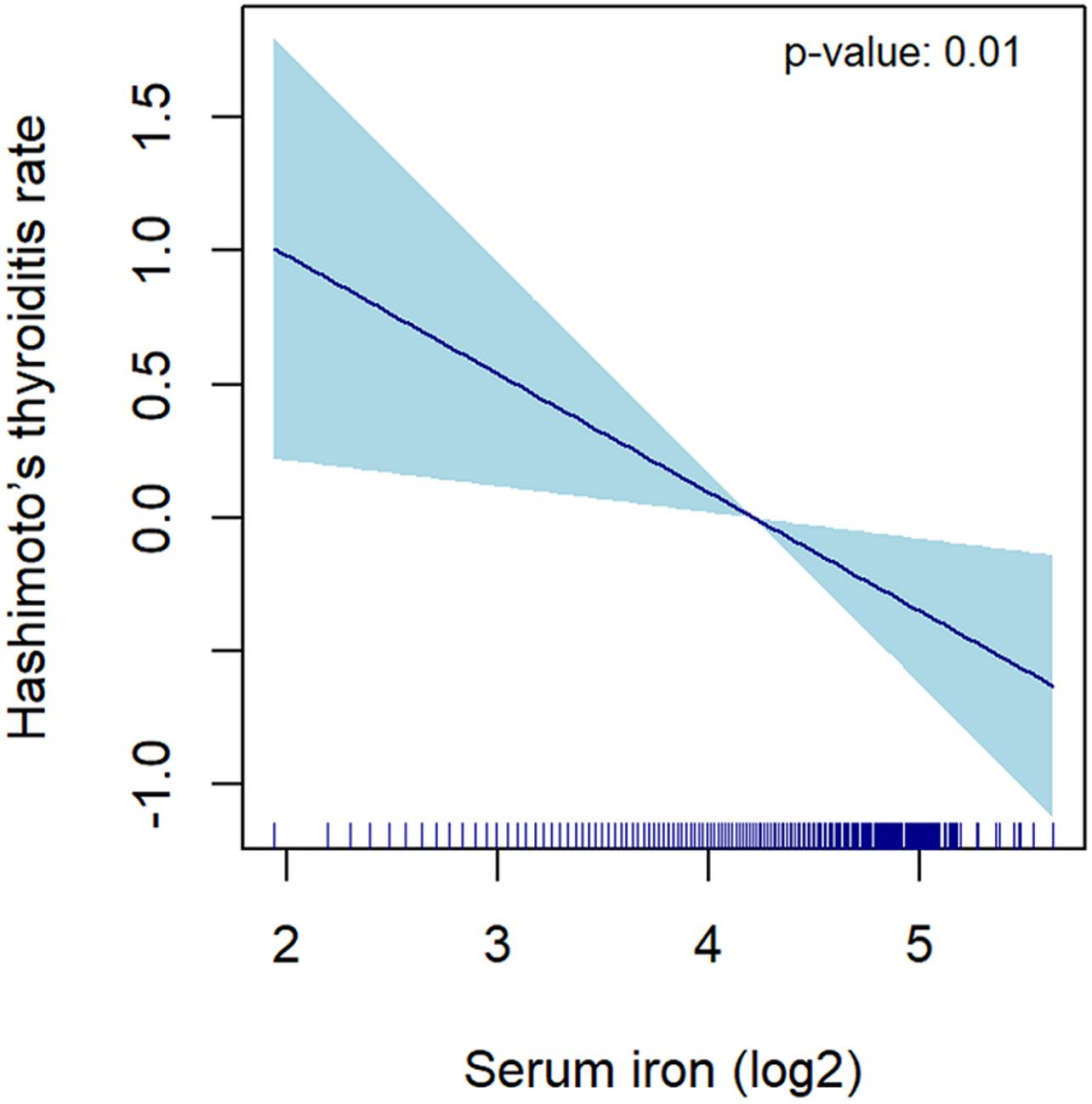
The correlation between serum iron (log2-transformed) and Hashimoto’s thyroiditis. The dark blue line represents the smooth curve fit between variables. The light blue area represents the 95% CI of the fit. Age, race/ethnicity, education level, marital status, poverty income ratio, BMI, alcohol intake, smoking, FT3, FT4, TSH, physical activity, hypertension, diabetes, Iodine, Calcium were adjusted.

For the sensitivity analysis, the continuous variable of serum iron was segmented into four equal parts, which was done to affirm the stability and precision of the findings. The results for the categorical variable were consistent with the effects observed for serum iron as a continuous variable, as indicated by the *p*-values from the trend test (Table 2). Smooth curves were drawn, adjusting for covariates in Model 3, to further examine the potential linear association between serum iron levels and HT. The log2-transformed serum iron showed a linear correlation with HT (Figure 4). These findings indicate an inverse relationship between serum iron levels and the risk of HT.

### 3.6 The relationship between serum iron and TPOAb and TgAb

HT is marked by the generation of antibodies that target antigens in the thyroid, and iron plays a crucial role in TPO function. Accordingly, an examination was also conducted on the association between serum iron and both TPOAb and TgAb (Table 3). Participants with higher serum iron levels showed a decrease in TPOAb levels, which is in line with Models 1, 2, and 3. In Model 3, serum iron (log2-transformed) was found to have a negative correlation with TPOAb levels (*β* = −15.47; 95% CI -25.01, −5.92; *p* = 0.003), but no significant association was observed with TgAb levels (*β* = −3.52; 95% CI -9.35, 2.30; *p* = 0.225). Hence, for the purpose of sensitivity analysis, we transformed the continuous variable into quartiles. Compared to participants in the lowest quartile (Q1), those in the highest quartile (Q4) were significantly associated with lower TPOAb levels across all models, while no such association was found for TgAb levels.

**Table 3:** The association between serum iron and thyroid peroxidase antibodies, thyroglobulin antibodies.

Exposure	Model 1 OR (95% Cl)	*p* value	Model 2 OR (95% Cl)	*p* value	Model 3 OR (95% Cl)	*p* value
TPOAb						
Serum Iron (log2 transformation)	−8.90(−15.42, −2.37)	0.009	−9.80(−16.43, −3.17)	0.005	−15.47(−25.01, −5.92)	0.003
Q1(1.61,3.91)	Reference		Reference		Reference	
Q2(3.93,4.26)	3.57(−11.28,18.42)	0.631	3.21(−11.97,18.39)	0.671	3.53(−19.39,26.46)	0.754
Q3(4.28,4.56)	0.90(−12.42,12.21)	0.893	0.36(−12.85,13.57)	0.957	−0.24(−17.63,17.15)	0.978
Q4(4.57,6.06)	−11.99(−21.14, −2.84)	0.011	−12.94(−22.11, −3.77)	0.007	−20.30(−31.63, −8.98)	0.001
*p* trend	0.019		0.011		0.005	
TgAb						
Serum Iron (log2 transformation)	−2.88(−6.61,0.84)	0.126	−3.36(−7.10,0.39)	0.078	−3.52(−9.35,2.30)	0.225
Q1(1.61,3.91)	Reference		Reference		Reference	
Q2(3.93,4.26)	−1.97(−14.15,10.21)	0.747	−2.19(−14.77,10.38)	0.727	1.76(−11.91,15.44)	0.793
Q3(4.28,4.56)	−4.66(−15.15,5.83)	0.376	−5.44(−16.53,5.65.57)	0.328	−7.77(−19.35,3.81)	0.180
Q4(4.57,6.06)	−3.88(−13.18,5.42)	0.405	−4.46(−13.70,4.79)	0.336	−3.32(−15.35,8.70)	0.575
*p* trend	0.288		0.206		0.300	

Model 1: adjusted for none. Model 2: age and race/ethnicity were adjusted. Model 3: age, race/ethnicity, education level, marital status, poverty income ratio, BMI, alcohol intake, smoking, FT3, FT4, TSH, physical activity, hypertension, diabetes, Iodine, Calcium were adjusted.

**Figure 5:**
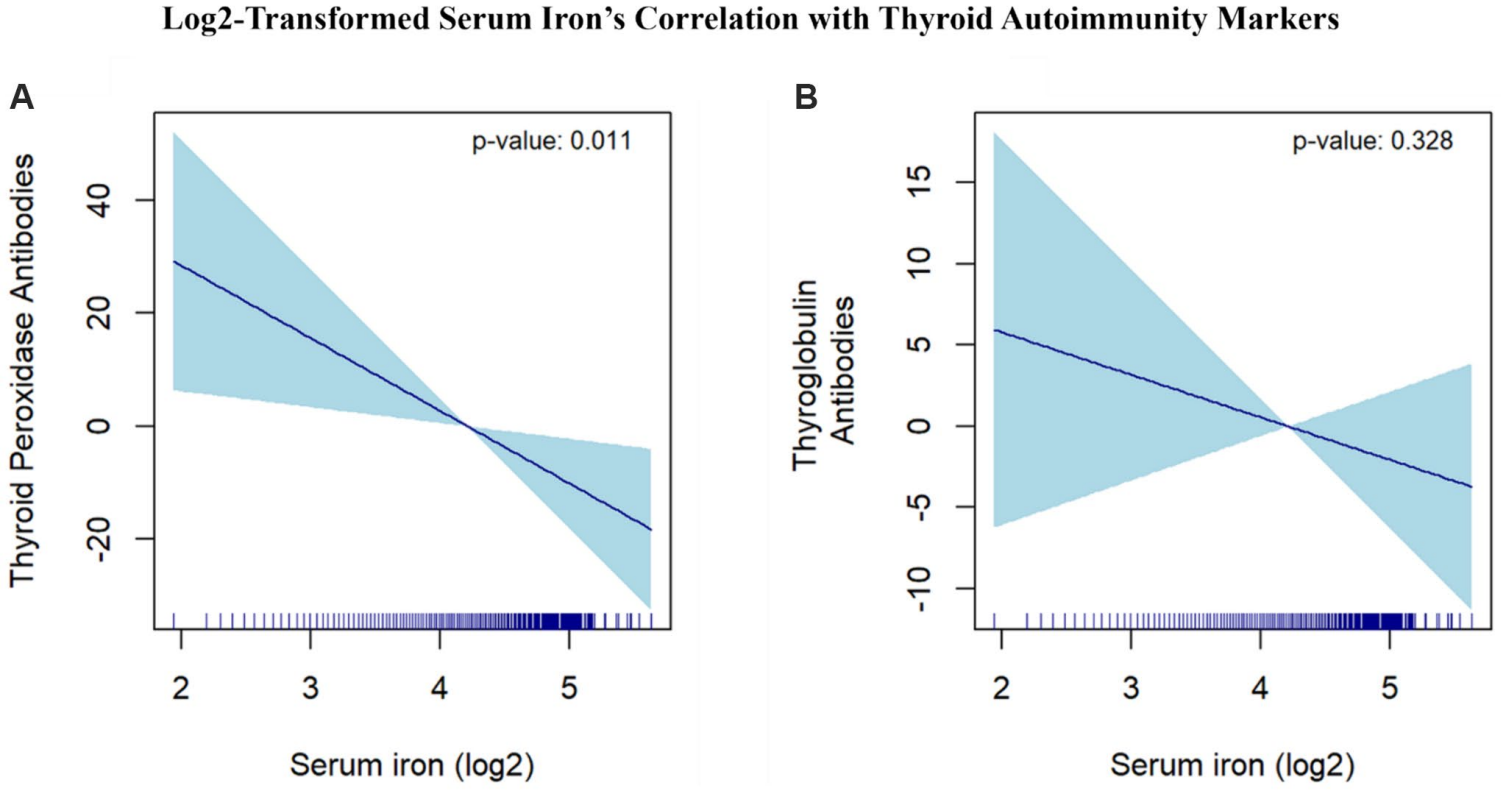
Log2-Transformed Serum Iron’s Correlation with Thyroid Autoimmunity Markers. **(A)** The correlation between serum iron (log2-transformed) and thyroid peroxidase antibodies. **(B)** The correlation between serum iron (log2-transformed) and thyroglobulin antibodies. The dark blue line represents the smooth curve fit between variables. The light blue area represents the 95% CI of the fit. Age, race/ethnicity, education level, marital status, poverty income ratio, BMI, alcohol intake, smoking, FT3, FT4, TSH, physical activity, hypertension, diabetes, Iodine, Calcium were adjusted.

We utilized smooth curve fitting to delve into potential non-linear associations between serum iron and the levels of TPOAb and TgAb. Smooth curves were formulated based on the fully adjusted model. The results indicated that serum iron has a linear negative association with TPOAb levels. Conversely, a curvilinear association was observed between serum iron and TgAb levels (Figure 5).

## 4 Discussion

HT predominantly affects women, particularly during their reproductive years (8–10, 37). Iron deficiency, commonly observed among HT patients, significantly impacts thyroid function due to iron’s essential role in thyroid hormone synthesis (15). To date, there have been no extensive, high-quality cross-sectional studies probing the association between serum iron levels and HT in females of reproductive age. Our study leverages data from the NHANES survey conducted between 2007 and 2012, focusing on a representative sample of United States females aged 15 to 44 years. Through this comprehensive cross-sectional analysis, we aimed to investigate the association between serum iron levels and HT. Our findings reveal an inverse relationship between serum iron levels and HT, suggesting that lower serum iron levels may contribute to the pathogenesis of this autoimmune thyroid disorder. This aligns with existing literature that underscores the importance of adequate iron levels for maintaining thyroid function and preventing HT (15, 38).

Specifically, each additional unit of serum iron was associated with a 43% decrease in the risk of HT (OR: 0.574; 95% CI: 0.572 to 0.576). A smooth curve analysis further highlighted the critical role of serum iron in HT prevalence among reproductive-aged females. Clinically, HT is primarily diagnosed by the presence of TPOAb and TgAb, with TPOAb being the most sensitive marker, as it has a positivity rate exceeding 95% among HT patients (35, 39). Our study found a negative linear correlation between TPOAb levels and HT (*β*: -15.47; 95% CI: −25.01 to −5.92), while the relationship between TgAb and HT was more complex and curvilinear. The exact mechanism linking iron deficiency to TPOAb positivity, but not TgAb positivity, remains uncertain. One hypothesis suggests that iron deficiency may induce post-translational modifications in TPO, potentially creating new epitopes or revealing previously hidden ones, thereby enhancing TPO’s immunogenicity. Another theory posits that reduced TPO activity due to iron deficiency may contribute to an increase in thyroid autoimmunity (40).

Iron plays a crucial role in thyroid function, primarily as a cofactor for TPO, where it is a central atom in the prosthetic groups of the enzyme’s active site (15, 41). In the body, iron is mainly contained in hemoglobin and myoglobin, but a small portion is also present in various cytochromes and other hemoproteins, including TPO (41). Iron deficiency can impair TPO activity, leading to decreased production of thyroid hormones T3 and T4, which disrupts thyroid function and may trigger compensatory increases in TSH levels. Elevated TSH can promote thyroid tissue inflammation and contribute to the autoimmune processes observed in HT (15, 17–19, 28, 38, 39). Furthermore, iron deficiency is associated with increased oxidative stress and inflammation (42), both of which can exacerbate thyroid autoimmunity by enhancing the production of thyroid autoantibodies, particularly TPOAb (40). Our findings reinforce the biological plausibility that lower serum iron levels may be associated with HT, emphasizing that adequate iron is essential for optimal thyroid function. These mechanisms underscore the importance of maintaining adequate iron levels to prevent or mitigate the risk of HT, especially in women of reproductive age, who are more prone to iron deficiency and thyroid autoimmunity.

The clinical implications of our findings are substantial. Given the high prevalence of iron deficiency among patients with HT, it is crucial to integrate routine screening for iron deficiency into clinical practice. Identifying low serum iron levels should prompt further investigation into potential underlying conditions that impair iron absorption, such as celiac disease or autoimmune gastritis, which can exacerbate thyroid dysfunction (43). Addressing these conditions can improve iron status and, consequently, support thyroid health. Iron supplementation, particularly with well-tolerated formulations like ferrous bisglycinate, should be considered to alleviate deficiency and enhance thyroid function (44). Our findings underscore the importance of comprehensive nutritional assessments in HT management and suggest that monitoring and managing iron levels can be beneficial. Incorporating iron management into clinical guidelines for HT could enhance treatment outcomes, reduce autoantibody levels, and improve overall thyroid function.

Given that dietary patterns and healthcare practices may have changed over time, it is crucial for future studies to replicate these analyses with more recent data and account for contemporary factors such as changes in dietary habits and public health interventions. A recent analysis of NHANES data highlights significant changes in mineral intake over time, further underscoring the necessity of using updated data in future research (45). Our findings may also be relevant to populations in different geographic and cultural contexts. Studies from Egypt (46) and China (47) have shown associations between iron deficiency and thyroid disorders, suggesting that our results could be applicable across diverse settings. However, regional differences in diet, genetics, and environmental factors should be considered. Future research should validate our findings in various populations to confirm their broader applicability. It is worth noting that previous studies have reported mixed results regarding the association between iron levels and thyroid function, potentially due to differences in populations, severity of iron deficiency, or assessment methodologies (43, 48–50). Our study contributes by specifically examining this relationship in reproductive-aged women, offering valuable insights into the complex interaction between iron status and thyroid autoimmunity.

In this study, we controlled for several potential confounders, including age, race, education, marital status, BMI, and lifestyle factors. However, it is important to recognize that other variables, such as dietary iron intake, gastrointestinal conditions affecting absorption, and gut microbiota composition, may also influence serum iron levels and thyroid function (51). While our analysis accounted for various known confounders, factors like dietary habits and chronic diseases were not included. Research suggests that dietary iron intake and type (heme vs. non-heme), as well as gut microbiota diversity, can significantly impact iron absorption and bioavailability (52–54). Given that iron is crucial for many pathogens and gut bacteria (55–58), future studies should explore these factors to better understand the relationship between serum iron levels and HT.

Further prospective studies and clinical trials are needed to explore the mechanisms linking iron deficiency to HT, particularly in reproductive-aged women, as our study highlights. Longitudinal data focusing on this demographic will help clarify the temporal relationship between iron status and thyroid autoimmunity, providing insights into how these interactions influence HT development and progression. Understanding these relationships in this specific population will guide targeted prevention and treatment strategies, ultimately improving clinical care for women of reproductive age who are vulnerable to iron deficiency and thyroid dysfunction. By advancing this line of research, we can better address the unique needs of reproductive-aged women with HT, enhancing their overall health outcomes.

## 5 Conclusion

In essence, our study uncovers an inverse association between serum iron levels and HT in females of reproductive age. Serum iron levels were found to be negatively correlated with TPOAb levels and exhibited a non-linear relationship with TgAb levels. These findings emphasize the potential role of serum iron in HT pathogenesis and underscore the need for future prospective studies to validate and further explore these relationships.

## Data Availability

The datasets presented in this study can be found in online repositories. The names of the repository/repositories and accession number(s) can be found in the article/supplementary material.
